# The Right Anterior Thoracotomy Approach to Resect a Cardiac Papillary Fibroelastoma of the Aortic Valve

**DOI:** 10.7759/cureus.7136

**Published:** 2020-02-28

**Authors:** Besir Topal, Vikash Hindori, Sjaak Pouwels, Robert Riezebos

**Affiliations:** 1 Cardiothoracic Surgery, Onze Lieve Vrouwe Gasthuis (OLVG), Amsterdam, NLD; 2 Surgery, Haaglanden Medisch Centrum, The Hague, NLD; 3 Cardiology, Onze Lieve Vrouwe Gasthuis (OLVG), Amsterdam, NLD

**Keywords:** minimally invasive cardiac surgery, right anterior thoracotomy, papillary fibroelastoma, cardiothoracic surgery, cardiac tumors

## Abstract

A cardiac papillary fibroelastoma (CPFE) is reported to be the second most common cardiac neoplasm after myxoma cordis. CPFEs are histologically benign, frequently asymptomatic, but highly thrombogenic, which could lead to systemic and peripheral embolization. We present a case of a 68-year-old-patient, with a history of angioosteohypertrophy syndrome, who presented at our emergency department (ED) with symptoms of transient ischemic attacks. A thorough investigation, including echocardiography, revealed a neoplasm on the left coronary cusp (LCC) of the aortic valve. The neoplasm was resected via a valve-sparing shave via the right anterior thoracotomy (RAT). The pathological assessment confirmed it to be CPFE. CPFE is a rare but treatable cause of thromboembolism. The removal of CPFEs has classically been performed through a full median sternotomy. We like to present the first case of a valve-sparing removal of a CPFE on the aortic valve through a RAT approach.

## Introduction

The cardiac papillary fibroelastoma (CPFE) is the second most common cardiac neoplasm following myxoma [1]. CPFE are histologically benign tumors and mostly asymptomatic. They may involve the aortic, mitral, and tricuspid valves as well as the left ventricular outflow tract. Clinically, manifestation includes symptoms of transient ischaemic attacks, stroke, heart failure, myocardial infarction, and cardiac arrest [2]. Early surgical intervention is the treatment of choice to reduce the risk of recurrent embolism [3-7]. The postoperative prognosis is promising with no recurrences having been reported in the literature so far. Herein, we would like to present a case of a 68-year-old-patient, with a history of angioosteohypertrophy syndrome, who presented two times with the symptoms of transient ischaemic attacks and was found to have a CPFE on the left coronary cusp (LCC) of the aortic valve on echocardiography.

## Case presentation

**Figure 1:**
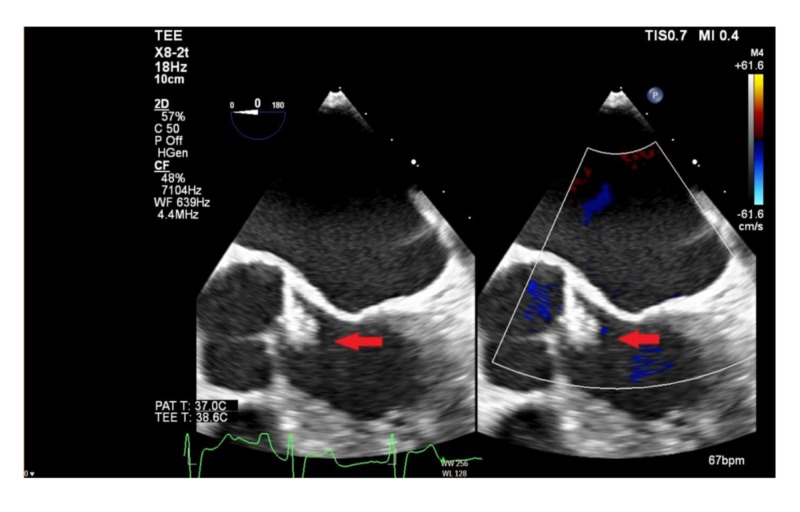
TTE/TEE images of CPFE (red arrow) of the LCC Left picture images without color flow adjustments; right with color flow adjustments TTE = Transthoracic Echocardiography; TEE = Transesophageal Echocardiography; CPFE = Cardiac Papillary Fibroelastoma; LCC = Left Coronary Cusp

A 68-year-old-patient, with a history of angioosteohypertrophy syndrome, was admitted to our hospital because of complaints of blurred vision and aphasia. The symptoms had already disappeared at the presentation. His vital parameters were normal with a blood pressure of 117/75 mmHg. Physical and neurologic examination showed no abnormalities. Laboratory findings, including complete blood count, glucose, and kidney and liver function tests were normal, except for slightly elevated serum cholesterol. The chest X-ray was normal, and the electrocardiography (ECG) showed normal sinus rhythm with a right bundle branch block (RBBB). Cerebral computed tomography angiogram (CTA) was negative for bleeding, ischemia, and any other intracranial pathology. Duplex ultrasonography of both carotid arteries was negative for significant stenosis or thrombosis. As part of the evaluation of an embolic stroke of undetermined source, transthoracic echocardiography (TTE) was performed, which revealed a mass of approximately 1 cm diameter on the LCC of the aortic valve. Consequent transoesophageal echocardiography (TEE) revealed a non-mobile mass on the LCC measuring 0,9 x 1 cm presumed to be a fibroelastoma or low-suspected vegetation (Figure 1). Coronary computed tomography showed normal coronary anatomy.

**Figure 2:**
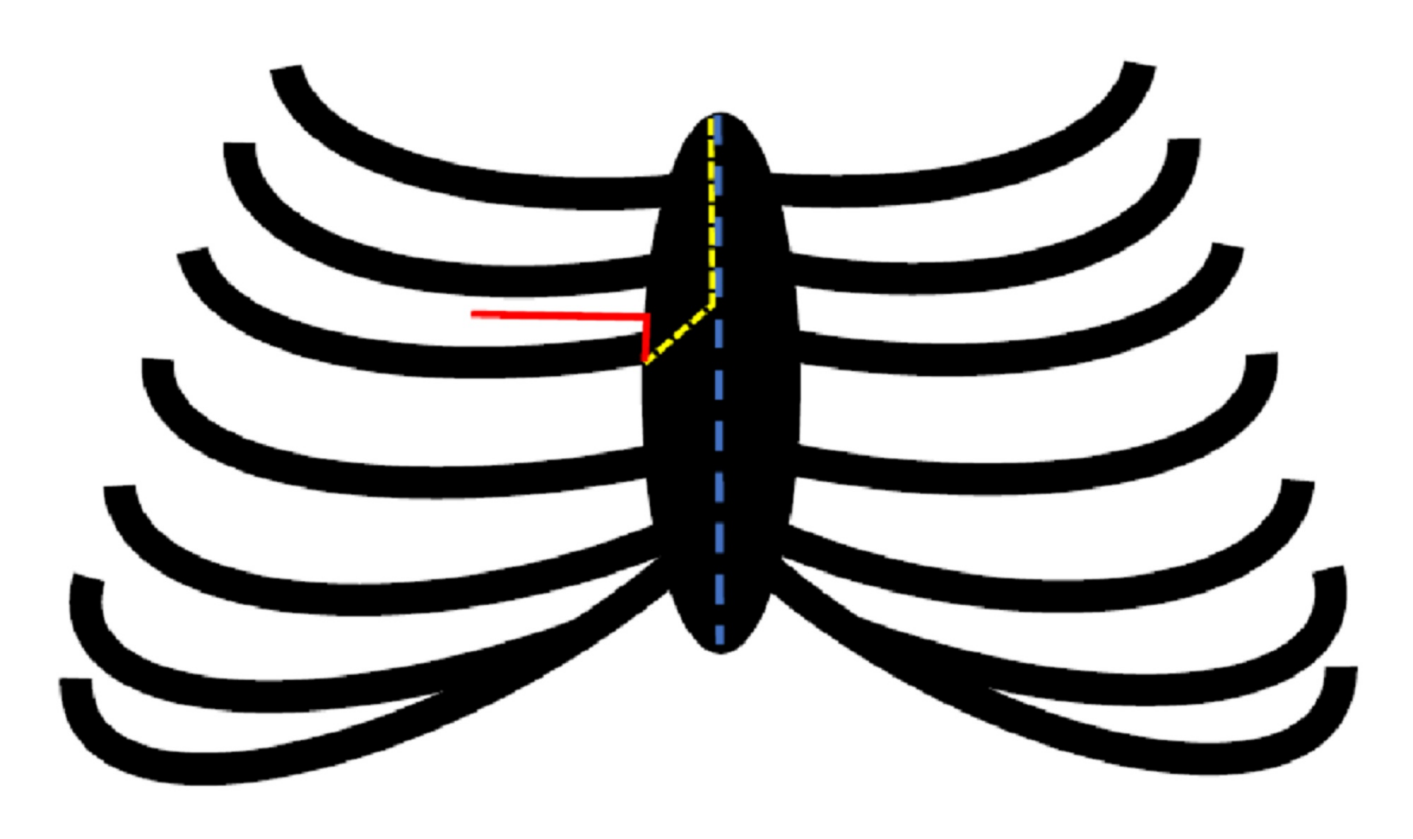
Schematic overview of operative approaches to reach the aortic valve 1 - (Blue Dotted Line) = (Conventional) Median Sternotomy; 2 - (Yellow Dotted Line) = Upper (Partial) Hemisternotomy (UHS); 3 - (Red Line) = Right Anterior Thoracotomy (RAT) (Figure developed and illustrated by B. Topal (MD) [8-9]

The patient underwent an aortic valve-sparing excision of tumorous tissue through a minimally invasive RAT approach. Figure 2 gives an overview of several operative approaches to reach the aortic valve.

The procedure is performed under general anesthesia and with the use of a double-lumen tube. Surgical access was performed through a 5 cm incision at the second intercostal space through which the pericardium is exposed, as described by Glauber et al. [8-9]. To increase visibility after deflating the right lung, the following was done. Further exposure is realized after transecting the third rib from the sternum, which is reattached at the end of the operation. In addition, a soft tissue retractor is used. After opening the pericardium 3 cm above the phrenic nerve, the aortic valve can be easily reached through an aortotomy after clamping the aorta. The procedure is done on an arrested heart and cardiopulmonary bypass, which is initiated through the cannulation of the femoral artery and vein. Complete CPFE excision on the LCC was performed under direct view and confirmed by echocardiography. After cardiopulmonary bypass, a TEE evaluation was performed to confirm total excision.

**Figure 3:**
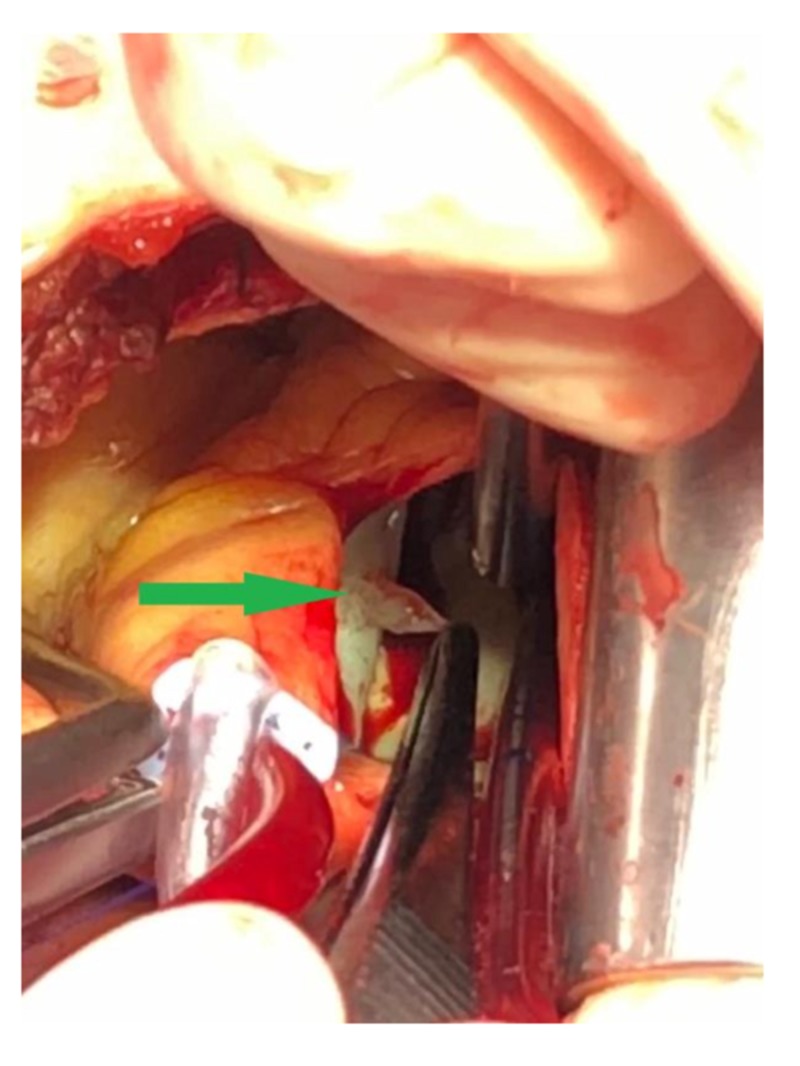
Intraoperative image of CPFE (green arrow) of the LCC CPFE = Cardiac Papillary Fibroelastoma; LCC = Left Coronary Cusp

Consistently, this patient was scheduled for further follow-up by clinical examination and TTE at our department for cardiology. Intraoperative exposition of the aortic valve showed particularly unimpaired leaflets with a small known mass at the ventricular side of the LCC and NCC, which were removed without complications (Figure 3). A pathological assessment of the resected specimen determined it to be a CPFE. Postoperative TEE showed no residual tumorous tissue and confirmed the total integrity of the native valve without aortic valve regurgitation. The patient's recovery was uneventful, and he could be discharged after three days.

## Discussion

Primary cardiac neoplasms are a rare entity with a prevalence of 0.2% - 0.28% among the general population. They are regularly benign and occur mostly in adult patients with a primarily male predominance (58%) [10-11]. Due to the low incidence of these neoplasms and the lack of longitudinal surveillance, their natural history remains unclear.

Historically, most findings of cardiac neoplasms were based on autopsy studies; currently, with modern echocardiography, such neoplasms are increasingly diagnosed in vivo [12-13]. Most CPFEs are located on the left side of the heart and are generally asymptomatic. Although histologically benign, the existence of CPFE is potentially dangerous due to its high thrombogenicity. There is an increased risk of systemic embolization into coronaries, systemic circulation, and pulmonary circulation. The risk depends on the size, mobility, and location of the CPFE [14]. It is essential to differentiate CPFEs from myxomas, thromboemboli, endocarditis, and calcification because of the differences in treatment.

CPFEs are typically located on the valvular sides of the heart, commonly involving the aortic and mitral valves [15]. Surprisingly, despite this valvular location, valvular dysfunction or regurgitation is rare [16]. Left-sided CPFEs and valvular locations are more prone to embolic events [17].

In the assessment of CPFE, TTE and TEE are the only imaging modalities required preoperatively, except for cases where the presence of coronary artery disease must be assessed. Coronary artery angiography (CAG) for coronary evaluation is associated with increased risk because the catheter may dislodge a fragment and may cause systemic embolism [18-19]. This reasoning was in accordance with our above-mentioned case. CPFEs located on the aortic valve will need a cardiac computed tomography angiography (CCTA) to evaluate the coronary anatomy and avoid the aforementioned increased risk of embolism [20].

Therapeutic evaluation should take into account the existing symptoms and the risk of life-threatening systemic embolization. There is a general agreement that symptomatic patients should be referred for surgery because complete resection of CPFEs is curative with a sublime postoperative prognosis. CPFEs rarely recur after excision, thus it is regarded as safe and curative to shave off the tumor, as we did in our case. Asymptomatic patients with a left-sided tumor, with a size >1 cm, should be treated surgically due to the increased risk of systemic embolism and sudden death [20]. On the other hand, asymptomatic patients with left-sided, non-mobile neoplasms could be followed using echocardiographic surveillance until the onset of symptoms, tumor enlargement, or increased mobility [20]. In this way, TTE/TEE proves to be a valuable imaging technique for surveillance and decision-making. Symptomatic patients who are not candidates for surgery should be treated with long-term anticoagulation, although there is a lack of randomized controlled trials to support this method [20].

Minimally invasive cardiac surgery has been reported to improve patient outcomes, including less blood loss, fewer transfusions, less pain, shorter hospitalization, and earlier return to work [8-9]. Our patient had no perioperative complications and was discharged three days after surgery. In our experience, the right anterior thoracotomy approach is an effective and safe surgical technique with an improved patient outcome as compared to a full median sternotomy.

## Conclusions

A valve-sparing shave of CPFE via the RAT approach seems to be an effective and safe surgical technique with an improved patient outcome as compared to a full median sternotomy.

## References

[1] Gowda RM, Khan IA, Nair CK, Mehta NJ, Vasavada BC, Sacchi TJ (2003). Cardiac papillary fibroelastoma: a comprehensive analysis of 725 cases. Am Heart J.

[2] Da Fontoura Tavares CM, De Oliveira NA, Miguel R, Atie J (2004). Recurrent ventricular fibrillation secondary to aortic valve tumor. Heart Rhythm.

[3] Soca R, Illatopa V, Cutipa M, Aguilar C (2017). Multiple papillary fibroelastomas in a patient with severe mitral stenosis. J Card Surg.

[4] Xie XJ, Yu FX, Liu HD (2017). Incidental papillary fibroelastoma of the tricuspid valve. J Card Surg.

[5] Arikan AA, Omay O, Aydin F (2017). Aortic valve replacement for papillary fibroelastoma. J Card Surg.

[6] Wickendon J, Khan H, Chaubey S, Butt S, Desai J. (2016). Papillary fibroelastoma causing left ventricular outflow obstruction and systolic anterior motion (SAM). J Card Surg.

[7] McCanny A, Imran Hamid U, Carroll S, Jeganathan R (2017). Fibroelastoma of the aortic valve. J Card Surg.

[8] Glauber M, Miceli A, Bevilacqua S, Farneti PA (2011). Minimally invasive aortic valve replacement via right anterior minithoracotomy: early outcomes and midterm follow-up. J Thorac Cardiovasc Surg.

[9] Glauber M, Miceli A, Gilmanov D, Ferrarini M, Bevilacqua S, Farneti PA, Solinas M (2013). Right anterior minithoracotomy versus conventional aortic valve replacement: a propensity score matched study. J Thorac Cardiovasc Surg.

[10] Jha NK, Khouri M, Murphy DM (2010). Papillary fibroelastoma of the aortic valve--a case report and literature review. J Cardiothorac Surg.

[11] Law KB, Phillips KR, Cusimano RJ, Butany J (2009). Multifocal "tapete" papillary fibroelastoma. J Clin Pathol.

[12] Cheitlin MD, McAllister HA, de Castro CM (1975). Myocardial infarction without atherosclerosis. JAMA.

[13] Sun JP, Asher CR, Yang XS (2001). Clinical and echocardiographic characteristics of papillary fibroelastomas: a retrospective and prospective study in 162 patients. Circulation.

[14] Mezilis NE, Dardas PS, Tsikaderis DD, Zaraboukas T, Hantas A, Makrygiannakis K, Anastasiadis K (2005). Papillary fibroelastoma of the cardiac valves: a rare cause of embolic stroke. Hellenic J Cardiol.

[15] Harling L, Athanasiou T, Ashrafian H, Kokotsakis J, Brown V, Nathan A, Casula R (2012). Minimal access excision of aortic valve fibroelastoma: a case report and review of the literature. J Cardiothorac Surg.

[16] Butany J, Nair V, Naseemuddin A, Nair GM, Catton C, Yau T (2005). Cardiac tumours: diagnosis and management. Lancet Oncol.

[17] Georghiou GP, Vidne BA, Sahar G, Sharoni E, Fuks A, Porat E (2010). Primary cardiac valve tumors. Asian Cardiovasc Thorac Ann.

[18] Koniari I, Apostolakis E, Baikoussis NG (2009). eComment: cardiac papillary fibroelastoma: a current assessment. Interact Cardiovasc Thorac Surg.

[19] Kelpis TG, Ninios VN, Economopoulos VA, Pitsis AA (2010). Aortic valve papillary fibroelastoma: a three-dimensional transesophageal echocardiographic appearance. Ann Thorac Surg.

[20] Castillo JG, Silvay G (2010). Characterization and management of cardiac tumors. Semin Cardiothorac Vasc Anesth.

